# *trans*-3,3′,4,5′-Tetra­meth­oxy­stilbene

**DOI:** 10.1107/S160053681102575X

**Published:** 2011-07-09

**Authors:** Ri-An Yan, Xiao-Xia Li, Guo-Qiang Li

**Affiliations:** a Department of Food Science and Engineering, Jinan University, Guangzhou 510632, People’s Republic of China

## Abstract

The title compound, C_18_H_20_O_4_, was synthesized by a Wittig–Horner reaction of diethyl 3,4-dimeth­oxy­benzyl­phosphate and 3,5-dimeth­oxy­benzaldehyde. In the crystal, the dihedral angle between the two aromatic rings is 2.47 (12)°. All the meth­oxy groups are almost coplanar with the aromatic ring to which they are attached [C—C—O—C torsion angles = −2.8 (3), −5.2 (4), −176.3 (2) and −178.0 (2)°].

## Related literature

For the bioactivity of stilbene-based compounds, see: Nam *et al.* (2001 ▶); Belleri *et al.* (2005 ▶); Gosslau *et al.* (2005 ▶); Sale *et al.* (2004 ▶). For reference structural data, see: Piao *et al.* (2002 ▶); Shibutani *et al.* (2004 ▶).
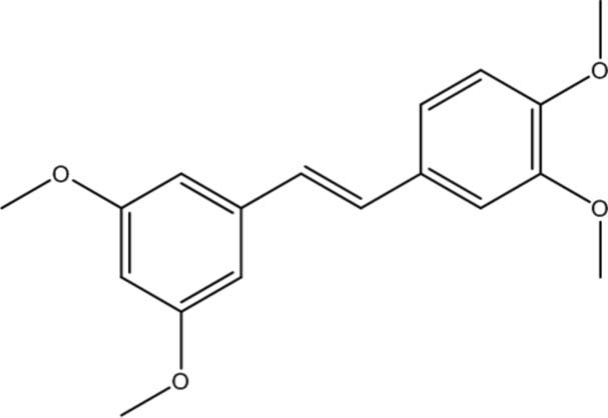


## Experimental

### 

#### Crystal data

C_18_H_20_O_4_
*M* *_r_* = 300.34
Orthorhombic, 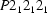

*a* = 5.2431 (2) Å
*b* = 11.9840 (7) Å
*c* = 25.6315 (11) Å
*V* = 1610.51 (14) Å^3^
*Z* = 4
Cu *K*α radiation
μ = 0.71 mm^−1^
*T* = 293 K
0.42 × 0.11 × 0.07 mm

#### Data collection

Agilent Xcalibur Sapphire3 Gemini ultra diffractometer
Absorption correction: multi-scan (*CrysAlis PRO*; Agilent, 2010 ▶) *T*_min_ = 0.781, *T*_max_ = 1.000
2952 measured reflections
2032 independent reflections
1791 reflections with *I* > 2σ(*I*)
*R*_int_ = 0.020

#### Refinement

*R*[*F* ^2^ > 2σ(*F* ^2^)] = 0.039
*wR*(*F* ^2^) = 0.095
*S* = 1.13
2032 reflections
203 parameters
H-atom parameters constrained
Δρ_max_ = 0.13 e Å^−3^
Δρ_min_ = −0.15 e Å^−3^

### 

Data collection: *CrysAlis PRO* (Agilent, 2010 ▶); cell refinement: *CrysAlis PRO*; data reduction: *CrysAlis PRO*; program(s) used to solve structure: *SHELXS97* (Sheldrick, 2008 ▶); program(s) used to refine structure: *SHELXL97* (Sheldrick, 2008 ▶); molecular graphics: *OLEX2* (Dolomanov *et al.*, 2009 ▶); software used to prepare material for publication: *OLEX2*.

## Supplementary Material

Crystal structure: contains datablock(s) global, I. DOI: 10.1107/S160053681102575X/ff2018sup1.cif

Structure factors: contains datablock(s) I. DOI: 10.1107/S160053681102575X/ff2018Isup2.hkl

Supplementary material file. DOI: 10.1107/S160053681102575X/ff2018Isup3.cml

Additional supplementary materials:  crystallographic information; 3D view; checkCIF report

## Figures and Tables

**Fig. 1.**
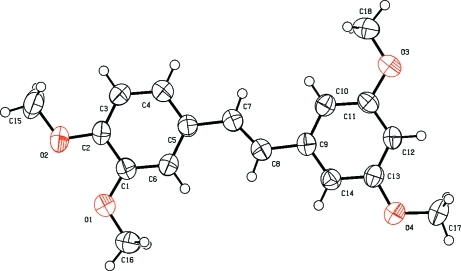
The molecular structure of the title compound with the atom numbering scheme. Displacement ellipsoids are drawn at the 30% probability level.

## supplementary crystallographic information

### Comment

Many stilbene-based compounds show important bioactivity, acting as anti-angiogenesis (Belleri *et al.*, 2005) and anti-cancer (Gosslau *et al.*, 2005; Sale *et al.*, 2004; Nam *et al.*, 2001) agents.

In the crystal struture of the title compound, the dihedral angle between the two aromatic rings is 2.47 (12)°. All methoxy groups are almost coplanar with their parent aromatic rings (torsion angles -2.8 (3)°, -5.2 (4)°, -176.3 (2)°, 178.0 (2)° for C10—C11—O3—C18, C12—C13—O4—C17, C2—C1—O1—C16, C1—C2—O2—C15, respectively).

### Experimental

Sodium methoxide (17.00g, 314.0mmol) was added to a well-stirred suspension of the diethyl 3, 4-dimethoxybenzylphosphate (33.00g, 114.0mmol) in dry DMF (130 ml) at 268K. After 30 min, the 3,5-dimethoxybenzaldehyde (11.00g, 66.0mmol) in dry DMF (60 ml) was added dropwise, and the reaction mixture was allowed to stir at room temperature for 12h. The mixture was then poured into ice-water. After filtration, the precipitate was collected as a yellow solid. Then the impure was recrystallized with ethanol to yield the title compound(m.p.339K, yield 59.1%). The product was dissolved in the mixture of ethyl acetate (15%) and petroleum ether (85%), colorless crystals suitable for X-ray analysis were obtained when the solution was exposed to air at room temperature for 6 d.

### Refinement

The C-bound H atoms were positioned geometrically and were included in the refinement in the riding-model approximation, with C—H distances 0.98 Å (CH_3_),*U*_iso_(H) = 1.5*U*_eq_(C); 0.95 Å (CH),*U*_iso_(H) =1.2*U*_eq_(C);

### Crystal data

**Table:**

C_18_H_20_O_4_	*D*_x_ = 1.239 Mg m^−^^3^
*M**_r_* = 300.34	Cu *K*α radiation, λ = 1.5418 Å
Orthorhombic, *P*2_1_2_1_2_1_	Cell parameters from 1273 reflections
*a* = 5.2431 (2) Å	θ = 3.5–62.6°
*b* = 11.9840 (7) Å	µ = 0.71 mm^−^^1^
*c* = 25.6315 (11) Å	*T* = 293 K
*V* = 1610.51 (14) Å^3^	Needle, light colourless
*Z* = 4	0.42 × 0.11 × 0.07 mm
*F*(000) = 640	

### Data collection

**Table:**

Agilent Xcalibur Sapphire3 Gemini ultra diffractometer	2032 independent reflections
Radiation source: Enhance Ultra (Cu) X-ray Source	1791 reflections with *I* > 2σ(*I*)
mirror	*R*_int_ = 0.020
Detector resolution: 16.0288 pixels mm^-1^	θ_max_ = 62.7°, θ_min_ = 3.5°
ω scans	*h* = −3→5
Absorption correction: multi-scan (*CrysAlis PRO*; Agilent, 2010)	*k* = −13→11
*T*_min_ = 0.781, *T*_max_ = 1.000	*l* = −17→29
2952 measured reflections	

### Refinement

**Table:**

Refinement on *F*^2^	Primary atom site location: structure-invariant direct methods
Least-squares matrix: full	Secondary atom site location: difference Fourier map
*R*[*F*^2^ > 2σ(*F*^2^)] = 0.039	Hydrogen site location: inferred from neighbouring sites
*wR*(*F*^2^) = 0.095	H-atom parameters constrained
*S* = 1.13	*w* = 1/[σ^2^(*F*_o_^2^) + (0.0429*P*)^2^] where *P* = (*F*_o_^2^ + 2*F*_c_^2^)/3
2032 reflections	(Δ/σ)_max_ = 0.005
203 parameters	Δρ_max_ = 0.13 e Å^−^^3^
0 restraints	Δρ_min_ = −0.15 e Å^−^^3^

### Special details

**Table:**

Experimental. CrysAlisPro, Agilent Technologies (2010). Empirical absorption correction using spherical harmonics, implemented in SCALE3 ABSPACK scaling algorithm.
Geometry. All esds (except the esd in the dihedral angle between two l.s. planes) are estimated using the full covariance matrix. The cell esds are taken into account individually in the estimation of esds in distances, angles and torsion angles; correlations between esds in cell parameters are only used when they are defined by crystal symmetry. An approximate (isotropic) treatment of cell esds is used for estimating esds involving l.s. planes.
Refinement. Refinement of F^2^ against ALL reflections. The weighted R-factor wR and goodness of fit S are based on F^2^, conventional R-factors R are based on F, with F set to zero for negative F^2^. The threshold expression of F^2^ > 2sigma(F^2^) is used only for calculating R-factors(gt) etc. and is not relevant to the choice of reflections for refinement. R-factors based on F^2^ are statistically about twice as large as those based on F, and R- factors based on ALL data will be even larger.

### Fractional atomic coordinates and isotropic or equivalent isotropic displacement parameters (Å^2^)

**Table:**

	*x*	*y*	*z*	*U*_iso_*/*U*_eq_	
O2	0.2225 (4)	0.53444 (16)	0.41963 (6)	0.0686 (5)	
O3	0.4129 (3)	0.05952 (15)	0.78118 (6)	0.0590 (5)	
O4	−0.2862 (3)	0.29517 (15)	0.82410 (6)	0.0604 (5)	
O1	−0.1078 (4)	0.59508 (15)	0.48866 (6)	0.0628 (5)	
C4	0.3989 (5)	0.3431 (2)	0.52589 (10)	0.0555 (6)	
H4	0.5169	0.2873	0.5332	0.067*	
C5	0.2240 (5)	0.3738 (2)	0.56362 (8)	0.0481 (6)	
C7	0.2275 (5)	0.3164 (2)	0.61401 (9)	0.0533 (6)	
H7	0.3621	0.2674	0.6196	0.064*	
C8	0.0604 (5)	0.3265 (2)	0.65256 (9)	0.0519 (6)	
H8	−0.0766	0.3742	0.6467	0.062*	
C10	0.2460 (5)	0.1874 (2)	0.71574 (9)	0.0475 (6)	
H10	0.3631	0.1637	0.6908	0.057*	
C18	0.5886 (5)	0.0176 (2)	0.74345 (11)	0.0649 (7)	
H18A	0.4962	−0.0122	0.7143	0.097*	
H18B	0.6904	−0.0402	0.7589	0.097*	
H18C	0.6974	0.0770	0.7318	0.097*	
C14	−0.1071 (5)	0.3027 (2)	0.74094 (8)	0.0467 (5)	
H14	−0.2284	0.3567	0.7329	0.056*	
C17	−0.3087 (6)	0.2439 (3)	0.87367 (9)	0.0762 (9)	
H17A	−0.3381	0.1654	0.8693	0.114*	
H17B	−0.4491	0.2764	0.8923	0.114*	
H17C	−0.1542	0.2551	0.8931	0.114*	
C13	−0.1035 (5)	0.25517 (19)	0.79054 (8)	0.0467 (6)	
C12	0.0729 (5)	0.17448 (19)	0.80283 (9)	0.0476 (6)	
H12	0.0755	0.1430	0.8360	0.057*	
C2	0.2307 (5)	0.4775 (2)	0.46577 (8)	0.0505 (6)	
C3	0.4011 (5)	0.3941 (2)	0.47741 (9)	0.0562 (6)	
H3	0.5193	0.3717	0.4525	0.067*	
C6	0.0495 (5)	0.4586 (2)	0.55153 (8)	0.0489 (6)	
H6	−0.0698	0.4803	0.5764	0.059*	
C1	0.0516 (5)	0.51025 (19)	0.50366 (9)	0.0470 (6)	
C11	0.2464 (4)	0.14063 (19)	0.76539 (9)	0.0458 (5)	
C16	−0.2818 (5)	0.6360 (2)	0.52633 (11)	0.0659 (7)	
H16A	−0.3802	0.6956	0.5115	0.099*	
H16B	−0.1893	0.6632	0.5560	0.099*	
H16C	−0.3939	0.5769	0.5370	0.099*	
C15	0.4072 (7)	0.5055 (3)	0.38112 (10)	0.0841 (10)	
H15A	0.3910	0.5548	0.3518	0.126*	
H15B	0.3797	0.4300	0.3700	0.126*	
H15C	0.5751	0.5124	0.3957	0.126*	
C9	0.0682 (5)	0.27030 (18)	0.70361 (8)	0.0449 (5)	

### Atomic displacement parameters (Å^2^)

**Table:**

	*U*^11^	*U*^22^	*U*^33^	*U*^12^	*U*^13^	*U*^23^
O2	0.0870 (13)	0.0752 (13)	0.0434 (9)	0.0076 (12)	0.0101 (9)	0.0135 (9)
O3	0.0593 (10)	0.0559 (10)	0.0617 (10)	0.0104 (10)	−0.0042 (9)	0.0068 (9)
O4	0.0648 (10)	0.0701 (11)	0.0464 (9)	0.0110 (10)	0.0090 (8)	0.0026 (9)
O1	0.0669 (11)	0.0672 (11)	0.0542 (10)	0.0155 (11)	0.0045 (9)	0.0121 (9)
C4	0.0592 (15)	0.0524 (14)	0.0548 (14)	0.0072 (14)	0.0026 (14)	0.0046 (13)
C5	0.0507 (13)	0.0497 (14)	0.0439 (12)	0.0002 (13)	0.0001 (11)	0.0043 (11)
C7	0.0600 (15)	0.0520 (15)	0.0478 (13)	0.0057 (13)	−0.0002 (12)	0.0050 (12)
C8	0.0566 (13)	0.0502 (14)	0.0490 (13)	0.0045 (13)	0.0000 (12)	0.0045 (12)
C10	0.0483 (13)	0.0482 (13)	0.0459 (13)	−0.0053 (12)	0.0028 (11)	−0.0017 (11)
C18	0.0560 (14)	0.0570 (16)	0.0816 (18)	0.0096 (15)	0.0003 (15)	−0.0009 (15)
C14	0.0504 (13)	0.0438 (12)	0.0459 (12)	0.0006 (12)	−0.0013 (11)	0.0014 (11)
C17	0.090 (2)	0.093 (2)	0.0449 (14)	0.0152 (19)	0.0171 (14)	0.0093 (15)
C13	0.0501 (13)	0.0501 (14)	0.0399 (12)	−0.0048 (12)	−0.0010 (11)	−0.0025 (11)
C12	0.0527 (13)	0.0527 (14)	0.0374 (11)	−0.0015 (13)	−0.0032 (11)	0.0042 (11)
C2	0.0609 (15)	0.0519 (14)	0.0388 (11)	−0.0039 (14)	0.0018 (11)	0.0050 (11)
C3	0.0622 (14)	0.0581 (15)	0.0484 (13)	0.0069 (14)	0.0117 (13)	0.0010 (13)
C6	0.0515 (13)	0.0535 (14)	0.0417 (12)	−0.0013 (12)	0.0044 (11)	0.0008 (12)
C1	0.0506 (14)	0.0462 (13)	0.0443 (12)	0.0005 (12)	−0.0026 (11)	0.0034 (11)
C11	0.0460 (12)	0.0415 (12)	0.0499 (12)	−0.0013 (12)	−0.0086 (11)	0.0014 (11)
C16	0.0627 (16)	0.0653 (18)	0.0697 (16)	0.0142 (15)	0.0000 (14)	−0.0005 (15)
C15	0.108 (2)	0.095 (2)	0.0490 (14)	−0.002 (2)	0.0240 (17)	0.0076 (16)
C9	0.0501 (13)	0.0434 (13)	0.0413 (12)	−0.0072 (12)	−0.0032 (11)	0.0021 (11)

### Geometric parameters (Å, °)

**Table:**

O2—C2	1.366 (3)	C18—H18C	0.9600
O2—C15	1.425 (3)	C14—H14	0.9300
O3—C18	1.427 (3)	C14—C13	1.393 (3)
O3—C11	1.368 (3)	C14—C9	1.383 (3)
O4—C17	1.416 (3)	C17—H17A	0.9600
O4—C13	1.374 (3)	C17—H17B	0.9600
O1—C1	1.371 (3)	C17—H17C	0.9600
O1—C16	1.416 (3)	C13—C12	1.375 (3)
C4—H4	0.9300	C12—H12	0.9300
C4—C5	1.382 (3)	C12—C11	1.383 (3)
C4—C3	1.385 (3)	C2—C3	1.373 (3)
C5—C7	1.463 (3)	C2—C1	1.407 (3)
C5—C6	1.403 (3)	C3—H3	0.9300
C7—H7	0.9300	C6—H6	0.9300
C7—C8	1.326 (3)	C6—C1	1.374 (3)
C8—H8	0.9300	C16—H16A	0.9600
C8—C9	1.472 (3)	C16—H16B	0.9600
C10—H10	0.9300	C16—H16C	0.9600
C10—C11	1.391 (3)	C15—H15A	0.9600
C10—C9	1.398 (3)	C15—H15B	0.9600
C18—H18A	0.9600	C15—H15C	0.9600
C18—H18B	0.9600		
			
C2—O2—C15	117.2 (2)	C12—C13—O4	124.8 (2)
C11—O3—C18	117.51 (19)	C12—C13—C14	120.4 (2)
C13—O4—C17	117.9 (2)	C13—C12—H12	120.3
C1—O1—C16	117.27 (19)	C13—C12—C11	119.3 (2)
C5—C4—H4	119.5	C11—C12—H12	120.3
C5—C4—C3	121.0 (2)	O2—C2—C3	124.9 (2)
C3—C4—H4	119.5	O2—C2—C1	115.9 (2)
C4—C5—C7	119.0 (2)	C3—C2—C1	119.2 (2)
C4—C5—C6	118.1 (2)	C4—C3—H3	119.6
C6—C5—C7	122.9 (2)	C2—C3—C4	120.7 (2)
C5—C7—H7	116.3	C2—C3—H3	119.6
C8—C7—C5	127.4 (2)	C5—C6—H6	119.4
C8—C7—H7	116.3	C1—C6—C5	121.2 (2)
C7—C8—H8	116.5	C1—C6—H6	119.4
C7—C8—C9	127.0 (2)	O1—C1—C2	114.9 (2)
C9—C8—H8	116.5	O1—C1—C6	125.4 (2)
C11—C10—H10	120.3	C6—C1—C2	119.7 (2)
C11—C10—C9	119.4 (2)	O3—C11—C10	123.9 (2)
C9—C10—H10	120.3	O3—C11—C12	115.0 (2)
O3—C18—H18A	109.5	C12—C11—C10	121.1 (2)
O3—C18—H18B	109.5	O1—C16—H16A	109.5
O3—C18—H18C	109.5	O1—C16—H16B	109.5
H18A—C18—H18B	109.5	O1—C16—H16C	109.5
H18A—C18—H18C	109.5	H16A—C16—H16B	109.5
H18B—C18—H18C	109.5	H16A—C16—H16C	109.5
C13—C14—H14	119.8	H16B—C16—H16C	109.5
C9—C14—H14	119.8	O2—C15—H15A	109.5
C9—C14—C13	120.5 (2)	O2—C15—H15B	109.5
O4—C17—H17A	109.5	O2—C15—H15C	109.5
O4—C17—H17B	109.5	H15A—C15—H15B	109.5
O4—C17—H17C	109.5	H15A—C15—H15C	109.5
H17A—C17—H17B	109.5	H15B—C15—H15C	109.5
H17A—C17—H17C	109.5	C10—C9—C8	122.8 (2)
H17B—C17—H17C	109.5	C14—C9—C8	117.9 (2)
O4—C13—C14	114.8 (2)	C14—C9—C10	119.3 (2)
